# Bridging to Heart Transplantation from the Biventricular Pulsatile Berlin Heart EXCOR Assist Device Support in a Patient with Advanced End-Organ Failure

**Published:** 2015-10-27

**Authors:** Zumrut Tuba Demirozu, Deniz Suha Kucukaksu

**Affiliations:** *Sisli Florence Nightingale Hospital, Istanbul Bilim University, Istanbul, Turkey.*

**Keywords:** *Heart failure*, *Heart-assist devices*, *Heart transplantation*, *Infection*

## Abstract

Long-term mechanical circulatory support is a life-saving technology while briding to heart transplantation. It increases the quality of life and preserves end-organ function for patients with advanced heart failure. The number of patients with advanced heart failure scheduled for heart transplantation before comorbidities escalate is on the rise. However, the device function is complicated by the bleeding-thrombosis and infection paradigm, hence the interest in understanding device thrombosis and infection. We describe a 27-year-old man with idiopathic cardiomyopathy, advanced end-organ failure, and severe infection, who was bridged to heart transplantation after 8 months on the Berlin Heart EXCOR (Berlin Heart AG, Berlin, Germany) biventricular support. The patient was discharged from the hospital in the third postoperative week after the recovery of his end-organ functions. At 29 months’ post-transplantation follow-up, his last cardiac biopsy was grade 0, his ejection fraction was 60%, and he was enjoying a good quality of life.

## Introduction

Long-term mechanical circulatory support is a mandatory therapy for advanced heart failure patients to preserve the secondary end-organ functions and increase the quality of life while waiting for heart transplantation. The hepatic and renal functions are important for the survival of the patient after heart transplantation. Advanced hepatic and kidney failure compromises the survival of the patient and the success of the heart transplantation.^1^^, ^^2^

The most complicated adverse events are infection and bleeding-thrombosis with long-term use of big pulsatile pumps. Infection and thrombogenic complications may cause pump failure and malfunction, which needs the explantation of the pump or even re-implantation of a new pump for bridging to heart transplantation.

Destination left ventricular assist device (LVAD) therapy may become available in the future as an alternative to heart transplantation. Infectious complications at present, especially in the externally driven LVAD, right ventricular assist device, and biventricular assist device, have been the major cause of morbidity and mortality in mechanically supported patients. Factors such as critical status, driveline exit site, and pocket infection may result in an ascending bacterial colonization.^3^ Various dressing techniques, education of the patient and caregivers, and application of topical antibiotic solutions to the wound area may help to localize the infection.

We describe a 27-year-old man with idiopathic cardiomyopathy, advanced end-organ failure, and severe infection who was bridged to heart transplantation after 8 months on the Berlin Heart EXCOR (Berlin Heart AG, Berlin, Germany) biventricular support.

## Case

A 27-year-old man was admitted to another hospital with shortness of breath, dyspnea, and cough in May 2011. His echocardiographic studies documented dilated cardiomyopathy and ejection fraction (EF) of 15%. Mid August 2011, he was hemodynamically unstable and was in New York Heart Association (NYHA) class III-IV. The liver enzymes were elevated due to congestive cardiomyopathy. He was defibrillated 5 times, had cardiac resuscitation, and was intubated. After extubation, he was transferred to our hospital for further treatment in September 2011. 

The patient had hepatomegaly, splenomegaly, and ascites on admission to our hospital. His echocardiographic studies demonstrated EF of 15%, systolic pulmonary artery pressure of 49 mmHg, left ventricular end-diastolic diameter of 6.6 cm, and left ventricular end-systolic diameter of 5.5 cm. Cardiac catheterization revealed pulmonary capillary wedge pressure of 28 mmHg, cardiac index of 1.4 lt/min/m^2^, cardiac output of 2.74 lt/min, right ventricular stroke work index of 3 gm-m/m^2^/beat, and pulmonary vascular resistance of 145 dyn.s/cm^5^. He underwent implantable cardioverter defibrillator implantation. His elevated liver enzymes were due to ischemic hepatitis, for which he was followed up by the gastroenterology department. He was discharged from the hospital with maximum medical heart failure therapy.

**Figure 1:**
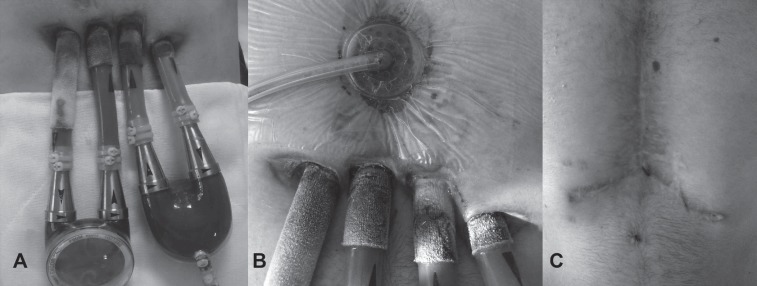
A) Berlin Heart EXCOR Biventricular Assist Device with four driveline exit sites B) Vacuum-assisted closure therapy applied at the lower sternal incision above the infected driveline exit sites C) Sternal incision status after heart transplantation

**Figure 2:**
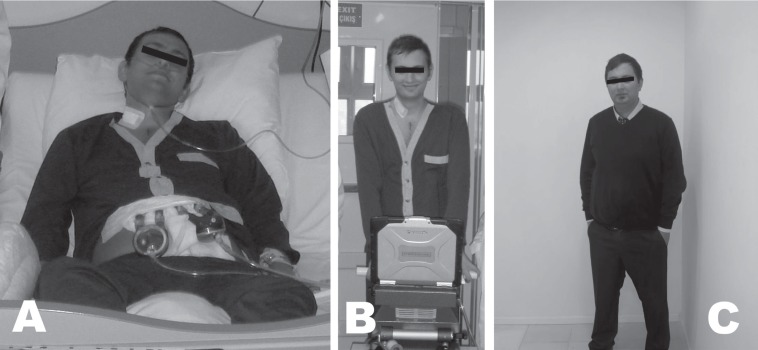
A) Patient with the Berlin Heart EXCOR Biventricular Assist Device in the third postoperative week in the cardiac surgery service B) Mobilization of the patient with the Berlin Heart EXCOR with his blue driving console C) Status of the patient over 29 months' follow-up post heart transplantation

One month later (October 2011), the patient was re-admitted to our hospital with decompensated heart failure. His liver enzymes and bilirubin levels were elevated 5 and, 8 times above the normal values, respectively. His renal function was deteriorated, and his urine output was < 30 cc/hr. He had both right and left advanced heart failure as well as end-organ dysfunctions. The patient was hospitalized in the Intensive Care Unit, where he was administered multidose inotropic medication. We deemed it advisable that he be listed as Status 1A while waiting for heart transplantation so that he could receive mechanical circulation support. He underwent the implantation of a Berlin Heart EXCOR (Berlin Heart AG, Berlin, Germany) biventricular assist device in December 2012. He was extubated on the 3rd postoperative day owing to multiple organ failure and transferred to the cardiac surgery service on the 7th postoperative day. His renal and hepatic functions started to recover in the 3rd postoperative week. He was listed as Status 1A at the national Organ Sharing System. The patient's big console precluded his discharge from the hospital. On the 55th postoperative day, he had purulent driveline infection. The antibiogram was studied for Methicillin-sensitive *Staphylococcus aureus* (MSSA). Treatment was commenced with Vancomycin and Rifampicin for more than 15 days. Driveline dressing was done separately for each driveline, and the debridement and curettage of the wound was performed before the application of a local antibiotic solution. The blood cultures were reported as *Staphylococcus aureus* and *Candida* species. He received anti-*Candida* medication until his blood cultures were reported negative. His lower sternal incision was tender, and purulent drainage was leaking from his driveline exit sites. It was decided to apply negative pressure wound therapy using the vacuum-assisted closure (VAC) method (Kinetic Concepts Inc., TX, USA). The VAC therapy dressing was changed every 3 days, and each driveline dressing was done separately with a topical antibiotic solution (Figure 1). By mid-June 2012, the patient had recovered from multiple organ failure and septic shock, and he was given Sulbactam/Ampicillin intravenously as an antibiotic. He had a heart offer and received plasmapheresis before undergoing heart transplantation in July 2012. The surgery went uneventfully, and he was transferred to the cardiac surgery ward, where he started to have cardiac rehabilitation on the 3rd postoperative day. He was discharged from the hospital in the 3rd postoperative week. At 29 months’ post-transplantation follow-up (Figure 2), his last cardiac biopsy was grade 0 and his EF was 60%. In addition, his quality of life showed an increase, and he was working as a finance consultant at a bank at the time of follow-up.

## Discussion

The prevalence of infection in the LVAD patients has been estimated at between 23% and 58%. Infection is a major mortal complication in patients with mechanical circulatory support. Multiple organ failure is a clinical entity frequently encountered in complicated status after the LVAD implantation. Positive blood cultures in multiple organ failure may be caused or aggravated by various factors.^2^^, ^^3^

Our patient developed driveline exit-site infection, a subcutaneous infection which needed debridement and curettage. The swab cultures obtained from the driveline exit site were important. Since our patient was listed as Status 1A, infection was crucial to the treatment plan, whereby he would be receiving immunsuppresion therapy after heart transplantation. It was crucial that he be hemodynamically stable and in good status with negative blood cultures. Our main focus was to limit the driveline exit-site infection so that it would be easy to manage the localized infection and prevent it from extending into the sternal cavity. Despite our efforts to control the infection and sepsis, there was always a risk for bleeding-thrombus formation. Since our patient could not survive without the biventricular assist device, we could not explant the pump if his infection was colonized inside the chest and around the pericardial sac. He could not tolerate a second operation like a pump exchange because of his borderline end-organ function. He had high panel-reactive antibodies (PRA) and needed immediate antibody removal with plasmapheresis before heart transplantation. Forest et al.^4^ reported that a large proportion (41%) of readmissions to the hospital in their study was for complications, most of them related to bleeding or infectious complications.

We believe that the appropriate timing of the LVAD implantation is important in patients with advanced heart failure to manage the secondary end-organ dysfunctions and also to increase the survival after heart transplantation. 

We herein described a young patient with idiopathic cardiomyopathy, advanced end-organ failure, and severe infection who was bridged to heart transplantation after 8 months on the Berlin Heart EXCOR biventricular support.

## Conclusion

The development of new techniques and strategies in mechanical circulatory support devices for the prevention of infection and bleeding will increase long-term survival and quality of life and also minimize post-implantation complications.

## Notes:

***This paper should be cited as: ***Demirozu ZT, Kucukaksu DS. Bridging to Heart Transplantation from the Biventricular Pulsatile Berlin Heart EXCOR Assist Device Support in a Patient with Advanced End-Organ Failure. J Teh Univ Heart Ctr 2015;10(4):201-204.
